# The Uneventful Use of Modern-Day Anesthetic Drugs in Post-polio Paralytic Patients: A Report of Three Cases

**DOI:** 10.7759/cureus.43913

**Published:** 2023-08-22

**Authors:** Joseph A Poonuraparampil, Habib Md R Karim, Pratyasha Nayak, Swati Vijapurkar, Gade Sandeep

**Affiliations:** 1 Anesthesiology, Manipal Tata Medical College, Manipal Academy of Higher Education (MAHE), Jamshedpur, IND; 2 Anesthesiology, Critical Care, and Pain Medicine, All India Institute of Medical Sciences, Deoghar, IND; 3 Anesthesiology, Critical Care, and Pain Medicine, All India Institute of Medical Sciences, Raipur, IND

**Keywords:** drug therapeutics, general anesthesia, spinal anesthesia, post-polio paralysis, scoliosis, poliomyelitis

## Abstract

The Western world has been polio-free for decades; however, many are affected by the stigmata of polio in several countries, including India. While general anesthesia (GA) and subarachnoid block (SAB) have been used successfully and widely, all those cases were mostly done with relatively older drugs and without additives. Therefore, further literature is needed to note the effect of newer anesthetic agents on post-polio paralytic patients for both GA, viz., propofol, fentanyl, rocuronium, and desflurane, and SAB with intrathecal hyperbaric bupivacaine and adjuvants. We report three male cases from Central India, in their 40s, with post-polio residual paralysis (PPRP), sarcopenia, and deformity of the lower limb and scoliosis; one case was managed under GA using desflurane-based low-flow anesthesia technique, and the other two under SAB, one with intrathecal fentanyl as an adjuvant to bupivacaine and the other without an adjuvant. The case series describes the effect of these modern-day anesthetic drugs and techniques.

## Introduction

Polio is a neuromuscular disorder affecting the neurons of the spinal cord's anterior horn, cerebellum nuclei, and cranial nerves, selectively damaging the motor nerves. It causes residual weakness and paralysis in the affected muscles of the neck, back, and hamstrings, leading to deformities of the limbs and spine, such as scoliosis and kyphosis [1]. Polio survivors can also develop new-onset muscle weakness, progressive in nature, and fatigue in the skeletal or bulbar muscles more than 15 years later, described as post-polio syndrome (PPS). Nevertheless, post-polio residual paralysis (PPRP) is a common happening. Although polio has been eliminated from most parts of the world, many polio survivors have post-polio deformities. Studies revealed increased potency of nondepolarizing muscle relaxants in remote poliomyelitis and PPS [2,3].

Nevertheless, data describing newer agents' dose-response characteristics in polio survivors with residual paresis are lacking. Considering possible altered sensitivity to anesthesia drugs, caution in the drug selection and dose is suggested [3]. Subarachnoid block (SAB) also poses concerns about exacerbating the neuromuscular disease process. Distorted anatomy and positioning difficulty predispose such patients to difficult spinal arachnoid puncture and outcome [4].

We present three cases of PPRP that required infraumbilical surgeries and were conducted under general anesthesia (GA) using balanced desflurane-based low-flow anesthesia and SAB using adjuvants. The report describes the effect of these newer anesthetic drugs on the duration of neuromuscular blockade, recovery from anesthesia, and motor deficits of PPS.

## Case presentation

Case I

A 37-year-old male, approximately 50 kg, was diagnosed with a fracture mid-shaft of the right femur following a road traffic accident (RTA) and planned for open reduction and internal fixation (ORIF) with titanium elastic nailing system (TENS). The patient had no history of head injury, nausea, vomiting, loss of consciousness, seizure, and ear, nose, and throat bleeding. No other injury or symptoms suggestive of respiratory or cardiac illness were noted. He gave a history of bilateral poliotic lower limbs with flexion contracture of the bilateral knee and hand-knee gait for 35 years. His Glasgow Coma Scale (GCS) was 15/15, heart rate (HR) was 90/minute, blood pressure (BP) was 134/80 mmHg, and respiratory rate (RR) was 18/minute. The bilateral knee had flexion contracture of approximately 90 degrees; muscle wasting and rigidity were present in both lower limbs. The right lower limb had a foot drop with an ankle power of 0/5. The left lower limb's ankle power was 1/5. Knee and hip power and deep tendon reflex (DTR) of the right lower limb could not be examined due to fracture, while DTR of the left lower limb's knee and ankle reflex was normal. The right lower limb had a loss of fine touch sensation. Airway examination did not predict anticipated difficulty. The spine was scoliotic of >50-degree Cobb's angle over (lumbar) L1-L3 levels with a tuft of hair on the skin at that area. Laboratory tests were within normal limits. Chest X-ray revealed scoliosis and within-normal-limit lung fields.

**Figure 1:**
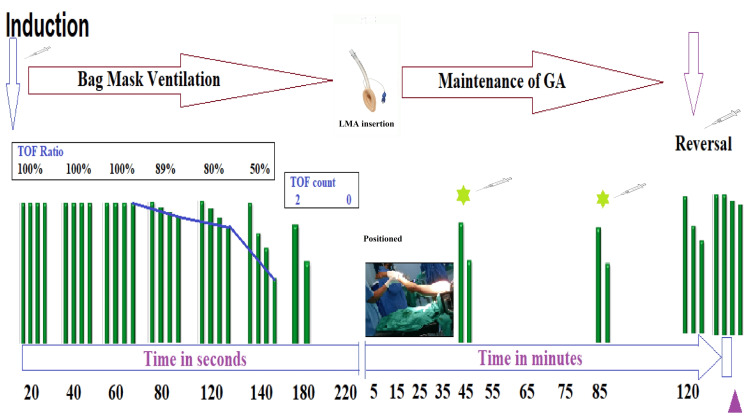
General anesthesia (GA) timeline with corresponding events and TOF monitoring results The star mark with the syringe indicates the top-up neuromuscular blockade drug (rocuronium) administration for maintenance. The purple triangle represents shifting the patient out of the operating room TOF, train of four; LMA, laryngeal mask airway

With informed consent, the patient was taken for surgery under GA. Following three minutes of preoxygenation, GA was induced with intravenous (IV) fentanyl 100 mcg and propofol 100 mg; 30 mg rocuronium IV was used as a muscle relaxant; the train of four (TOF) became zero at 220 seconds. At the same time, an age-adjusted minimum alveolar concentration (MACage) of 0.8 was achieved with 1:1 oxygen and nitrous oxide and 6% desflurane dial settings. Then, a classic laryngeal mask airway (LMA) of size 4 was inserted and secured, and volume control ventilation was initiated with a tidal volume of 400 mL, fraction of inspired oxygen (FiO_2_) of 50%, fresh gas flow of 800 mL/minute, RR of 12/minute, and positive end-expiratory pressure of 5 cmH_2_O. GA was maintained with 1:1 oxygen and nitrous oxide and titrated desflurane to maintain MAC of 0.8-1; rocuronium supplementation was TOF-guided. The patient was already on Ringer's lactate (RL) infusion, starting in the preoperative period, and has received 500 mL until the induction. The surgery lasted for two hours. IV RL 500 mL was intraoperatively administered for compensating blood loss of 150 mL, and urine output was 65 mL. Paracetamol 1 g and ondansetron 4 mg IV were also administered. At the end of the surgery, neuromuscular blockade was reversed with neostigmine 2.5 mg, and glycopyrrolate 0.4 mg IV was administered at TOF count 3 and was extubated (LMA removed) successfully without any complication or delay in recovery (Figure 1). His postanesthesia care unit (PACU) stay was one hour, and the ward stay was uneventful until discharge.

Case II

A 36-year-old male, 59 kg, was planned for transurethral incision of the prostate (TUIP). He suffered from poliomyelitis at the age of two years and developed progressive wasting and deformity of the right lower limb, including the foot. He could still walk without support and discomfort and perform daily activities independently. He had no other medical, surgical, or allergy-related co-morbidities or symptoms suggestive of cardiorespiratory illness. His HR was 75/minute, BP was 120/70 mmHg, and RR was 14/minute. Muscle wasting and foot drop were noted in the right lower limb; ankle power was 2/5. Sensations and DTR of the lower limbs were normal; anticipated airway difficulty was absent. Spine examination and an X-ray revealed mild scoliosis over L1-L4 levels; laboratory tests were within normal limits.

With informed consent, SAB was performed in the sitting position, using a 25-gauge Quincke needle via midline approach at the L3-L4 levels. Hyperbaric 0.5% bupivacaine 2.6 mL with fentanyl 25 mcg as adjuvant was injected intrathecally. The patient was made supine immediately, and the table was kept neutral; sensory and motor blockades were achieved within seven minutes. Intraoperative vitals remained within 20% of baseline. The patient received IV Ringer's lactate 200 mL as co-loading and 100 mL/hour as maintenance. The surgery lasted for 45 minutes. He was observed in the PACU; no surgical or anesthesia-related adverse events were noted. One hour into the PACU, the modified Bromage scale was grade 1, and he was shifted to the ward. Postoperative analgesia was maintained with IV paracetamol 1 g thrice a day for the first 24 hours and then converted to tablet paracetamol and discharged without complication.

Case III

A 36-year-old male, 54 kg, had a fall and sustained a fracture of the right distal supracondylar femur, inferior pole patella, and distal end of the left radius; ORIF of the distal right femur was planned. No history and clinical findings suggestive of head or other vital structure injuries were noted. He had no allergy to known drugs or medical and surgical co-morbidities. He suffered from polio in childhood, which resulted in right lower limb wasting and flexion contracture of 10 degrees at the knee, and he could walk only with support. Symptoms and signs of cardiorespiratory illness were absent; HR was 77 per minute, BP was 130/90 mmHg, RR was 16 per minute, and oxygen saturation (SpO_2_) was 98% on room air. Right lower limb foot drop was present; motor power could not be tested due to fracture, but sensations were intact. Airway examination and laboratory test reports were within normal limits.

With informed consent, he was taken up for surgery under SAB performed in the sitting position using a 24-gauge Quincke needle via midline approach at L3-L4 levels; hyperbaric 0.5% bupivacaine 3 mL was administered. The patient was made supine immediately, and the table was positioned at neutral; adequate sensory and motor blockades were achieved within four minutes. Intraoperatively, the patient had a fall of HR to 55 per minute but did not require any pharmacological intervention; blood pressure remained within a 20% fall from baseline. The patient received IV Ringer's lactate 250 mL as co-loading and 100 mL/hour as maintenance. The patient also received RL for blood losses at a rate of three times, and RL of nearly 900 mL was also infused. The surgery lasted for 120 minutes. He was observed in the PACU; no surgical or anesthesia-related adverse events were noted. One hour into the PACU, the modified Bromage scale was grade 1, and he was shifted to the ward. Postoperative analgesia was maintained with IV paracetamol 1 g thrice a day and IV tramadol 50 mg as and when required at pain score of >3 on a 0-10 rating scale for the first 24 hours and then converted to tablet paracetamol. He had no anesthesia-related complications until discharged from the hospital.

## Discussion

Poliovirus affects the central nervous system; the centers most severely affected are the brainstem and cerebellum [5]. It targets the anterior horn cells of the spinal cord during the early stages of paralytic poliomyelitis, leading to the loss of motor neurons and paralysis. The surviving neurons reinnervate the motor units by sprouting collaterals during healing. However, compared to the destroyed motor neurons, these new motor neurons are larger and fewer in number [3]. Anesthesia drugs, especially the neuromuscular blockade agents and intrathecally administered local anesthetics, are significantly related to neuromuscular junctions of the motor unit and spinal nerves, respectively. Therefore, the effect of such drugs on paralytic poliomyelitis and PPRP patients remains an area of further research, especially for newer agents used in current-day practice.

PPS presents 15-30 years after recovery from the initial acute paralytic poliomyelitis and presents with fatigue, myalgia, dysphagia, dysphonia, and difficulty breathing [6]. Particularly in individuals with pulmonary issues, neuromuscular blocking agents used in GA might cause problems if they are excessively used or incompletely reversed at the end of the surgery [3]. Suneel et al. revealed that post-polio patients have a low threshold for pancuronium and are prone to redevelop muscle weakness when exposed to susceptible drugs [7]. Thus, PPS and a history of poliomyelitis can be considered risk factors for increased muscle weakness in the postoperative period. A study suggested that patients with a remote history of polio have increased sensitivity to nondepolarizing muscle blockers [2]. Post-polio patients often have residual lesions involving the reticular activating system that may be of relevance to anesthesia. The reticular activating system is a theoretical site of action for most anesthetic agents, and such patients may show altered sensitivity to anesthetic drugs [3]. Therefore, patients with PPRP also appear to be at risk. For this reason, the selection of shorter-acting agents and the careful titration of doses to desired effect are required [3].

Recently, rocuronium has been used safely, but neostigmine reversal showed a delayed response, but subsequent sugammadex reversal on the same patient led to usual recovery [8,9]. We, however, noted the usual response to rocuronium and neostigmine reversal (Figure 1).

Historically, neuraxial anesthesia techniques have been a relative contraindication in patients with neurological disease, fearing the worsening of existing neurological dysfunction [10]. Polio is a neurological disease affecting the brain, spinal cord, and peripheral nervous system. Further worsening dysfunction and development of PPS in post-polio patients are known, making neuraxial anesthesia feared in these patients. However, case studies indicate the safe use of the technique without any intraoperative or postoperative adverse events [6,11]. Nevertheless, a recent report shows the occurrence of cauda equina syndrome in a PPS patient who underwent surgery under neuraxial anesthesia [12].

Reports using intrathecal fentanyl as an adjuvant are common but not reported or scarce in PPRP patients, as in our case. Though scoliosis in polio patients makes SAB a challenge and the prediction of sensory and motor blockades inaccurate, studies conducted on scoliotic spines have reported no adverse events in the time taken to reach complete block or recovery duration. Asymmetry in sensory and motor blockade spread is possible but not clinically significant. The correlation between Cobb's angle and the determination of the spread of spinal anesthetic in patients with scoliotic spines was given by Ballarapu et al., who concluded that failure of the SAB was more in patients who had Cobb's angle greater than 50 degrees, which may require assistance with advanced modalities such as ultrasound to reduce failure rate [13].

Moreover, body asymmetry makes positioning and SAB performance difficult [4]. Osteopenic limbs put them at risk for fracture. Muscle wasting, i.e., sarcopenia, poses a risk for peripheral nerve injury since the usual muscle mass may not protect the nerves. Autonomic dysfunction can result in intraoperative tachyarrhythmias and hemodynamic instability due to inflammation and scarring in the anterior horn extending to the intermediolateral columns where the sympathetic nerves travel. Intraoperative blood pressure was maintained within 20% of the baseline in both patients, with as minimal fluctuations as possible. Postoperative pain is also a concern in these patients. These patients have increased requirements for pain medications, and they should be titrated accordingly. Interestingly, our patient who received intrathecal adjuvant had a similar response pattern to that without adjuvant intraoperatively and slightly extended postoperative analgesia, as usually noted in other contemporary cases.

## Conclusions

Our case report indicates that desflurane-based low-flow anesthesia using rocuronium as a muscle relaxant led to the usual recovery. Even intrathecal fentanyl showed no notable complications or side effects in the immediate postoperative and perioperative course. Both patients who received spinal anesthesia also showed no worsening of motor function until hospitalization. However, only three cases cannot conclusively indicate safety; rather, it indicates that newer agents and adjuvants are probably safe, which needs to be assessed in the future with a larger sample.
